# Osteopathic Manipulative Medicine and Disorders: An Overview of Peer-Reviewed Publications 2018–2022

**DOI:** 10.7759/cureus.62185

**Published:** 2024-06-11

**Authors:** Cameron White, Yahui Xie, Jeremy Bigham, Ava Stanczak, David Ninan, Chien-An A Hu

**Affiliations:** 1 Department of Biomedical Sciences and Department of Osteopathic Manipulative Medicine, Kansas College of Osteopathic Medicine, Wichita, USA; 2 Department of Osteopathic Manipulative Medicine, Kansas College of Osteopathic Medicine, Wichita, USA; 3 Department of Biomedical Sciences and Department of Osteopathic Manipulative Medicine, Kansas College of Osteopathic Medicine, wichita, USA; 4 Department of Biomedical Sciences, Kansas College of Osteopathic Medicine, Wichita, USA

**Keywords:** omm research, vosviewer, evidence-based, peer-reviewed, osteopathic manipulative

## Abstract

Context

Osteopathic manipulative medicine (OMM) has been claimed to be effective in various human disorders and conditions. There have been many anecdotal claims to lend credence to the efficacy of this treatment modality. Recently, much work has been done in evidence-based, government-funded projects, and clinical trials in OMM research, and these studies have further demonstrated the efficacy of OMM as direct, integrated, or complementary mechanisms in treating various conditions.

Objectives

As the field of OMM research has grown significantly in the past few years, we set out to analyze the peer-reviewed publications on OMM in human disorders between January 2018 and December 2022.

Methods

We used keywords and terms which included “osteopath,” “osteopathic,” osteopathic manipulative medicine,” “osteopathic manipulative treatment,” and “disorder,” to systematically sample two public databases, PubMed and Science Direct. After the first query was recorded, we then applied more specific and stringent criteria to identify publications that (a) were written in English, (b) contained at least one human disorder/condition treated by OMM, (c) were co-authored by at least one osteopathic physician-scientist, and (d) contained at least one OMM technique.

Results

Our initial sampling of databases resulted in 404 publications. After applying our screening criteria, we identified and analyzed 249 (62%; 249/404) qualified publications in “OMM and Human Disorders” We then categorized them into (a) types of publications, (b) country origins of corresponding author(s), (c) groups of disorder and condition, (d) classification of the OMM used, and (e) relating the treated conditions with the five models of OMM. We found that in the 249 publications, 158 (63%) are research articles, 66 (27%) review papers, and 25 (10%) case reports. In addition, nine countries, the United States, Italy, Brazil, Spain, France, Germany, Canada, the United Kingdom (UK), and Australia contributed most of the publications of OMM. VOSviewer analysis identified a wide range of human disorders that were effectively treated with OMM. These included musculoskeletal, low back pain, neurological, headache, inflammation (including autoimmune conditions, COVID-19, lymphatic drainage), neonate/preterm infant disorders, anxiety, and dizziness.

Conclusions

Our comprehensive analysis showed that there has been a significant increase in peer-reviewed OMM publications in recent years, led by the United States osteopathic physician-scientists and European osteopathic scientists. OMM was found effective in treating not only common conditions such as pneumonia, low back pain, and musculoskeletal disorders, but also disorders such as inflammation, dizziness, headache, anxiety, and neonate/preterm infant disorders.

## Introduction

Osteopathic manipulative medicine (OMM) is a hands-on treatment modality that involves the manipulation of bones, joints, tissues, and muscles to assist in the diagnosis, prevention, and treatment of health conditions. In 1874, Dr. Andrew Taylor Still adopted the name osteopathic medicine (OM) to describe his new style of medical practice [1-2]. There are four tenets that establish the foundation for the whole-person approach of OMM: 1. The body is a unit; the person is a unit of body, mind, and spirit, 2. The body is capable of self-regulation, self-healing, and health maintenance, 3. Structure and function are reciprocally interrelated, 4. Rational treatment is based upon an understanding of the basic principles of body unity, self-regulation, and the interrelationship of structure and function. Thus, OMM is used to treat somatic dysfunction found throughout the body with the understanding of its capability of self-regulation and self-healing. The goal of this hands-on manipulation is to restore one’s natural function by allowing various aspects such as cardiovascular, respiratory, circulatory, musculoskeletal, and neurological systems to work together in unity [3,4]. It has been well demonstrated that OMM not only can serve as alternative, complementary, or facilitated treatment but also function as a direct treatment for human disorders such as pneumonia [5,6], low back pain [7-9], and musculoskeletal dysfunctions [4,10]. Although OMM is widely recognized both within the United States (US) and internationally, there has been controversy regarding the efficacy of OMM due to limited documentation of clear mechanisms of action, the quality of experimental designs, and the limited amount of evidence shown in the scientific literature [1,2,7,10]. This is due, in part, to the underrepresented and underfunded OMM research by the NIH and other government funding agents. According to the statistical analysis, Colleges of Osteopathic Medicine (COMs) currently educate approximately 34,000 future osteopathic physicians- 25% of all US student doctors. However, COMs receive only 0.1% of NIH funding, compared to 40% for allopathic medical institutions. This historic funding bias and disparity against OMM research needs to be addressed and resolved [2].

To bridge the disparities in the understanding of OMM and to identify other human disorders/conditions that OMM has been proven to be effective in treatment, we conducted a comprehensive literature review to examine the evidence-based, peer-reviewed publications published between January 2018 to December 2022. The elements included in this meta-analysis incorporate information regarding the publishing country, types of articles published, and mechanism of action, showcasing the efficacy of osteopathic manipulative treatment (OMT) for both healthcare professionals and the general population.

## Materials and methods

Literature Search and analyzing 

We queried two public scientific databases, NCBI PubMed (https://pubmed.ncbi.nlm.nih.gov) and ScienceDirect (https://www.sciencedirect.com) to obtain the sample articles. The combinations of keywords and terms we used in the research were “osteopath,” “osteopathic,” “osteopathic manipulative medicine,” “osteopathic medicine treatment,” “human disorder,” and “human condition.” We filtered the results to display only the 5 years between January 2018 and December 2022. To maintain integrity and focus on solely OMM, all the authors agreed to more specific and stringent criteria on publications that (a) were written in English, (b) contained at least one human disorder/condition treated by OMM, (c) were co-authored by at least one osteopathic physician or researcher, and (d) contained at least one OMT technique. We excluded publications that were educational, speculative, commentary, or discussion of usage rates of OMM in the osteopathic community. To stay focused on OMM, we also excluded other manual treatment modalities such as physical therapy, chiropractic care, acupuncture, and others. The relevant articles were then categorized by the nationality of the institution of the corresponding author, the date of publication, and the subject matter of the article. Furthermore, we also categorized and sorted the articles manually by subject matter using the described disorder/condition that was treated by OMT. We then identified eight specific categories to which most articles belonged: Musculoskeletal conditions, low back pain, neurological conditions, inflammation, neonate/preterm infant care, anxiety, vertigo/dizziness, and comprehensive reviews. Comprehensive review articles dealing with the entire discipline of OMM/OMT constituted their own category. Articles that were not topically related to these eight specific categories were placed into the “Other” category.

The Co-relationships between OMT and Disorders/Conditions by VOSviewer Analysis

To investigate the association and co-relationship between conditions and their treatments, we used the VOSviewer software (https://www.vosviewer.com) [11]. VOSviewer uses an artificial intelligence (AI) tool to construct and visualize bibliometric networks and generates a graphic map based on quantification and characteristics of individual keywords.

Upon launching of the VOSviewer application, we chose Create in the actions panel. Then selected, create a map based on bibliographic data which generates the map based on co-authorship, keyword co-occurrence, citation, bibliographic coupling, or co-citation. Then, subsequently clicked on read data from bibliographic databased files which supports the files: Web of Science, Scopus, Dimensions, Lens, and PubMed. Upon selection of the previously saved text file from PubMed, we selected co-occurrence for the analysis type, all keywords for the analysis unit, and full counting for counting method. We set the minimum of number of occurrences of a keyword to three for the threshold and kept the number of keywords to the number generated by the VOSviewer system. The last step consists of the verification of selected keywords. We manually verified the keywords to ensure relevance to OMM and accurate correspondence to publications retrieved from PubMed. We then generated the VOSviewer graphic map that grouped and linked the keywords by category and the strength of association between individual keywords. In addition, the graphic map also linked the conditions treated by OMM and their associated relationships with other disorders and conditions.

## Results

**Figure 1:**
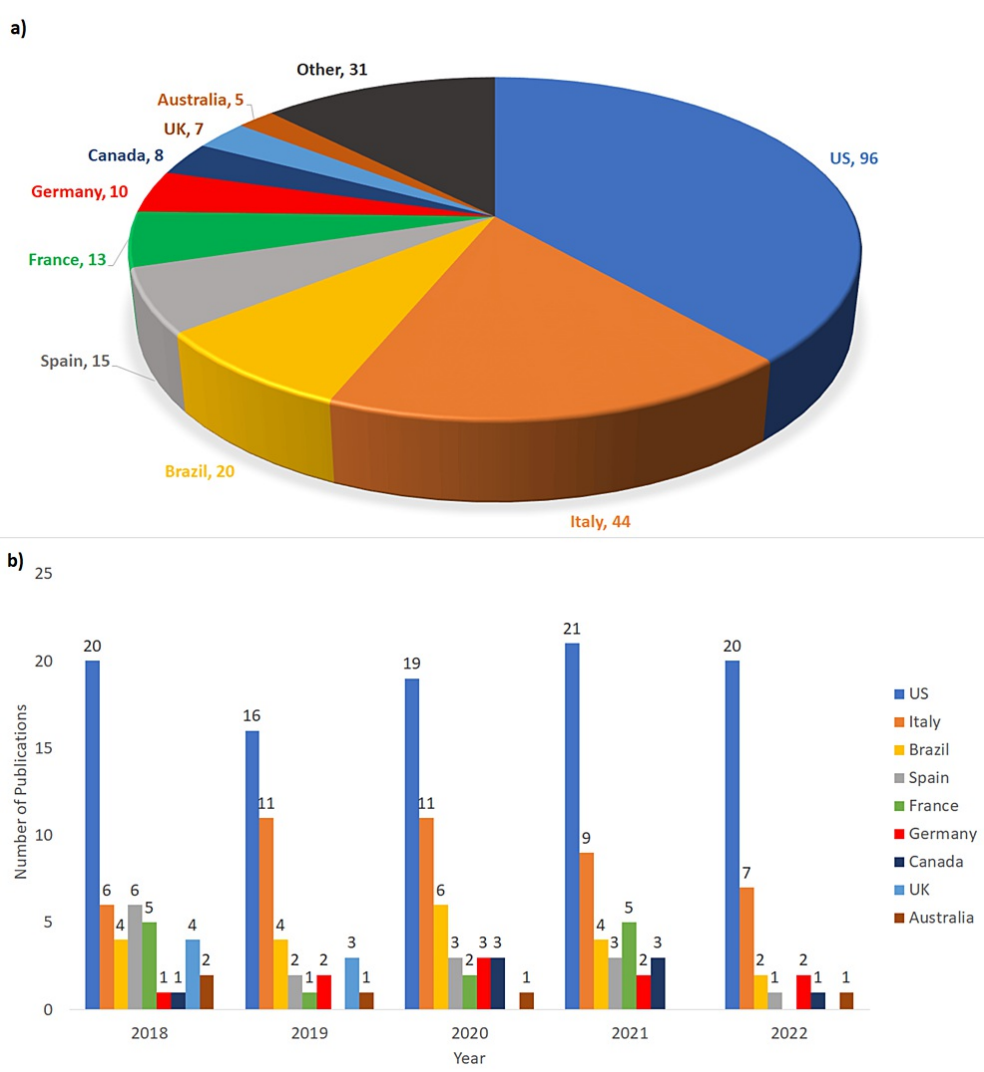
Peer-Reviewed Osteopathic Publications in Each Indicated Country Between 2018 and 2022. a) Total number of publications in nine countries, the US, Italy, Brazil, Spain, France, Germany, Canada, the United Kingdom (UK), and Australia from 2018-2022 were shown. The “Other” category contained the sum of other countries in the world that published only one or two papers. b) The retrieved 249 OMT articles over course of the study period were tracked according to the number of articles published in each year in the nine countries.

After screening the databases, retrieving the candidate publications, and further applying specific parameters for exclusion, we identified 249 publications that addressed OMM-treated disorders or conditions. To identify and characterize the research taking place in different countries, the publications were manually reviewed and categorized by the country of the corresponding author’s hosting institution(s). As shown in Figure 1A, the US has produced 96 papers (38.6%), Italy 44 (17.7%), Brazil 20 (8%), Spain 15 (6%), France 13 (5.2%), Germany 10 (4%), Canada 8 (3.2%), UK 7 (2.8%), and Australia 5 (2%). There were 20 other countries which had at least one publication, accounting for a total of 31 papers (12.5%). Another analysis was done regarding the number of publications published each year in the seven countries that had more than five publications over the entire study period. Each of the columns is the sum of the publications of the nine nations represented. We observed that in the US there was an increase in the number of publications until 2022, the rest of the world saw a decrease in publications in 2021 and 2022. This was possibly due to the COVID-19 pandemic (Figure 1B). Interestingly, about 38% (155/404) of the retrieved publications were educational, speculative, commentary, or a discussion of usage rates of OMM in the osteopathic community. However, these publications did not pertain to the goal of this study and were removed.

**Figure 2:**
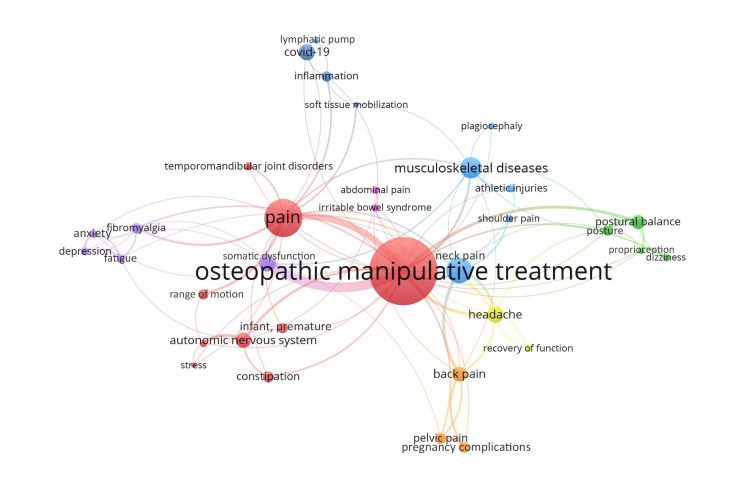
VOSviewer Analysis of Co-relationships and Co-occurrences of Keywords in the Sampled Osteopathic Manipulative Medicine (OMT) Articles. The titles and abstracts of the retrieved articles were analyzed through the artificial intelligence (AI)-assisted tool of VOSviewer. The size of the round circles (nodes) represents the number of times a keyword occurs in the published articles. The thickness of the lines indicates the strength of association between two different keywords. The color of the clusters is created based on association. Disorders and conditions that present with strong association are considered as within the same category.

**Figure 3:**
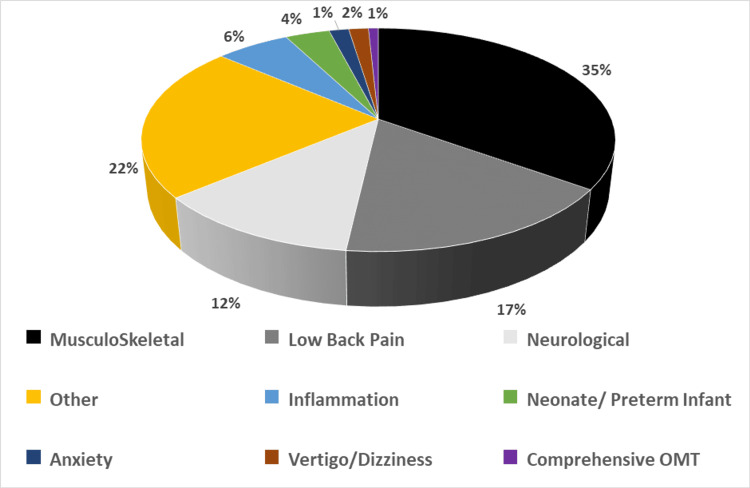
Overview of Topics of the Osteopathic Articles Surveyed. The graph showed the disorders and conditions treated by osteopathic manipulative medicine (OMM) in the sampled articles. Musculoskeletal, low back pain and neurological collectively comprised the traditional uses of OMM (Shown in black and gray) and comprised approximately two-thirds of the topics of the surveyed articles. Other fields highlighted were inflammation, anxiety, vertigo/dizziness, and neonatal/preterm infant.

Upon conducting a comprehensive review and VOSviewer analysis of the retrieved articles, the eight most common conditions treated by OMM were identified. They were anxiety, dizziness, headache, inflammation, low back pain, musculoskeletal dysfunction, neonate/preterm infant conditions, and neurological conditions (Figure 2). Low back pain, musculoskeletal conditions, and neurological conditions collectively comprised the traditional uses of osteopathic care (Figure 3) consisting of approximately two-thirds of topics of the surveyed articles. 

We further categorized the retrieved articles based on the specific disorders and conditions. The distribution was as follows: musculoskeletal disorders 35%, low back pain 17%, neurological disorders (including headache) 12%, inflammation (including autoimmune disorders, COVID-19, lymphatic drainage) 6%, neonate/preterm infant disorders 4%, anxiety 1.6%, and dizziness 1.6%. A miscellaneous category occupied 22% (Figure 3).

The mechanism of action and corresponding techniques for each disorder/condition have been identified to convey the efficacy of the treatment (Table 1). Among the eight most common human disorders treated by OMM, there were a diverse array of techniques used. Techniques used for frequently seen conditions were muscle energy, myofascial release, cranial manipulation, balanced ligamentous tension techniques, visceral manipulation, high-velocity low amplitude (HVLA), lymphatic pump, and occipito-atlantal decompression (OA-D). Treatment outcomes included reduction in frequency, intensity, severity, duration as well as prevention associated with disease progression.

## Discussion

This study examines evidence-based, peer-reviewed publications between 2018 to 2022 on “OMM and Human Disorders/Conditions” addressing the following questions: (1) Which countries contributed peer-reviewed papers in OMM research- what are the demographics? (2) What human disorders and conditions have been treated and researched by OMM- what is the co-relationship? (3) Which OMM techniques are used - the mechanisms? and (4) How can an integrated OMM model be used to treat the presented conditions-what treatments?

To answer each of these four questions, we began by showing the US has by far the most osteopathic publications of any individual country. However, it is only a plurality of the total research in the field. Given the historic association of the osteopathic profession in the US, it is surprising to see most the osteopathic research being conducted is outside of the US. Of particular interest are the major contributions of the osteopathic schools in Italy, publishing half as much research as the US even with fewer schools. Brazil is also of interest as an impressive number of papers were published despite relatively few institutions. Brazil has not been historically identified as a hub of osteopathic activity but as shown in Figure 1, it is one of the biggest contributors to the field. We were surprised by the amount of international contribution to the field of OM. To date, OM has been considered an American phenomenon. It is heartening to see that other countries have seen the advantages of such a treatment approach.

To answer the second and third questions, we analyzed several common disorders treated by OMM. Musculoskeletal disorders, low back pain, and neurological disorders collectively comprise the traditional uses of OM (Shown in black and gray, Figures 2-3) and comprise approximately two-thirds of the topics of the surveyed articles. Other fields highlighted are inflammation which includes autoimmune disorders, COVID-19, and lymphatic drainage, intervention in anxiety, and studies in neonatal/preterm infant disorders. Based on the findings of this comprehensive literature review and previously published literature [12-14], OMM is shown to be effective in treating not only common conditions such as chronic low back pain, dizziness, and musculoskeletal dysfunctions but also conditions like generalized anxiety disorder and preterm neonatal complications. All of these disorders benefit from regulation and healing provided by the manipulation of structure and function.

**Figure 4:**
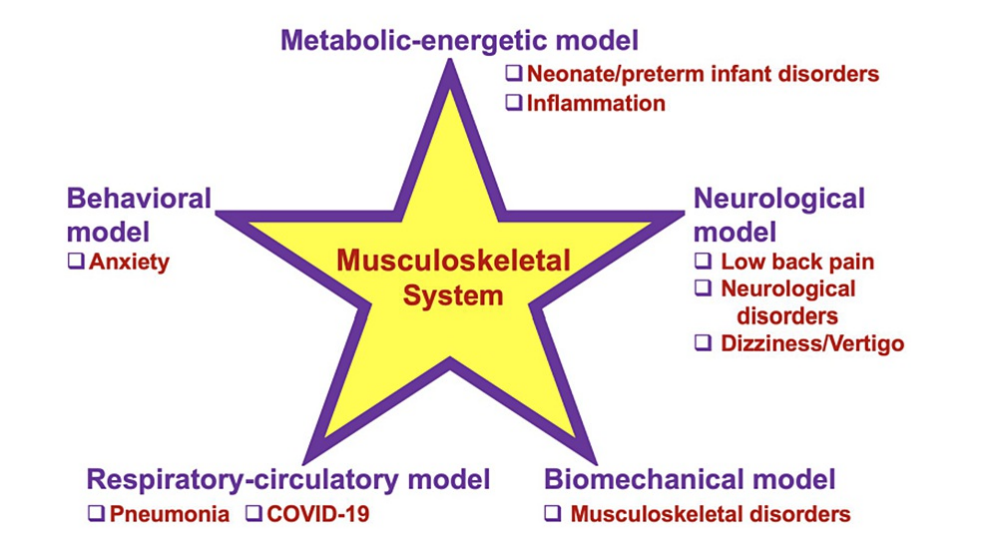
Viewing the Osteopathic Manipulative Medicine-Treated Disorders in the Context of the Five Models of Osteopathic Medicine. We made this summary figure to recapitulate the eight osteopathic manipulative medicine-treated disorders identified in this study in the context of the five models, metabolic-energetic, neurological, biomechanical, respiratory-circulatory, and behavioral. It is well accepted that the five models are interconnected by musculoskeletal anatomy and system.

To answer the fourth question, we listed the disorders of interest in the context of the six models of OMM (musculoskeletal, metabolic-energetic, biomechanical, respiratory-circulatory, neurological, and behavioral), each with its unique focus and value [1-3]. The musculoskeletal system appears at the center to demonstrate the interdependence of the models and this system (Figure 4). Each of the models is important for the complex interactions that occur throughout the body and demonstrate how a change in one system can cause a change in another system.

Our results also highlight the OMM techniques that are used to treat the disorders and conditions identified in this study (Figure 4, Table 1). For example, in the longitudinal PRECISION study conducted in a six-year period, OMM has been shown to improve pain intensity, pain impact, physical function, and health-related quality of life [7-9]. We examined the techniques and mechanism of action of most frequently seen patient discomforts (Table 1); anxiety, dizziness/vertigo, headache, inflammation, low back pain, musculoskeletal, neurological, and neonatal/preterm infant disorders.

Both cranial and articular technique have been successful in reducing severity, frequency and patient disability associated with dizziness [15]. OMM has also shown the ability to improve conditions caused by dysfunctional biochemical pathways in the human body, enabling a free pathway from neurovascular supply to the organs.

Findings show that OMM leads to a total reduction in both HAM-A Scores and Intolerance for Uncertainty Scale Scores in patients with generalized anxiety disorder. Treatment addresses the physical tension in one’s body, enabling a free pathway from neurovascular supply to the organs. Through the activation of β and C tactile receptors, OMM facilitates the release of oxytocin downregulating the hypothallus-pituitary adrenal (HPA) axis [16,17].

OMM also decreases circulating TNF-α reducing physical pain and progression of chronic diseases in patients with musculoskeletal disorders. Dysfunction in the musculoskeletal system can ultimately affect systemic function. Treating somatic dysfunction could decrease the risk and symptoms of other chronic diseases, such as diabetes mellitus [4,10].

In addition to the commonly known diseases treated using OMM, treatment targeting preterm infant complications has also been shown to be successful in reducing the length of stay of preterm infants [18-19]. Levels of biomarkers were higher in preterm compared to term infants, whereas IL-1b and IL-18 were lower [20]. Cytokines on the first day of life were higher in preterm infants born after complications associated with infections [21]. Preterm infants exhibit a diminished parasympathetic modulation of the heart [22].

Furthermore, cranial techniques have shown the ability to reduce pro-inflammatory substances and modulate parasympathetic and sympathetic actions of the heart affecting both inflammation and autonomic nervous system mechanisms [23,24]. The lymphatic pump has been shown to exert its effects on inflammation. Increasing the lymphatic drainage allows for an increase in thoracic duct lymph flow and in the passage of protein through the thoracic duct lymph. This leads to a decrease in the production of inflammatory markers such as NO2-, TNF-a , and IL-10, ultimately reducing inflammation and managing edema [25]. Given its clinical benefits, OMM has been utilized for treating COVID-19 patients in various settings [26,27]. This field is still new and full of challenges, further investigation is warranted to confirm the effectiveness and mechanism of treatment.

Spinal manipulation has been shown to benefit not only low back pain but also a wide range of neurological disorders. For example, radiculopathy, a condition caused by compression, inflammation, or damage to a spinal nerve root, often manifests as low back pain radiating down the leg. In the context of OMM, treating radiculopathy involves addressing the underlying causes of nerve root irritation through various techniques. The treatment primarily falls under the Neurological model but may also involve elements of the biomechanical models (Figure 4). While the Biomechanical and Neurological models are the primary approaches, integrating elements from other models, such as the respiratory-circulatory model, can provide a more comprehensive treatment plan. This holistic OMM approach can help address other contributing factors, such as circulation and metabolic health, ensuring a more effective and sustainable resolution of low back pain.” Alterations in cutaneous patterns of sympathetic activity affect the autonomic response in the human body. Modulation of alpha and gamma motor neuron activity as well as the neural plastic changes that alter net excitability exerts neuromuscular effects [28]. In addition, spinal manipulation has been shown to have hypoalgesia effects. For example, segmentation inhibition produced by the manipulation increases the stimulation of non-nociceptive A-b fibers which decreases the pain experienced by an individual. Temporal summation, which evokes pain, is also shown to decrease through spinal manipulation [29,30]. Through the impact of autonomic responses, neuromuscular and hypoalgesia effects, pain associated with neurological disorders are greatly reduced. Headache is one of the neurological conditions that have a significant impact on quality of life. OMM techniques such as myofascial release, muscle energy, HVLA, and occipito-atlantal decompression (OA-D) are shown to alleviate symptoms. OMM allows for regulation of metabolism, spinal and motor activity, and increased blood flow to the brain leading to a decrease headache frequency, intensity, and duration [23]. 

**Table 1:** Impact of OMM on Human Disorders and Conditions. OMM: osteopathic manipulative medicine

Condition	Mechanism of Action	OMT Technique	Outcome
Anxiety	Activation of β and C tactile receptors releasing oxytocin enables a free pathway from neurovascular supply to the organs [16]	Muscle energy; myofascial release cranial techniques; balanced ligamentous; tension technique; visceral manipulation	Total Reduction in Hamilton Anxiety Rating Scale (HAM-A) Scores Intolerance for Uncertainty Scale Scores
Dizziness/Vertigo	Normalize impaired structure-function relationships or homeostatic mechanisms [15]	Cranial technique; articular technique	Reduction in: Disability associated with dizziness, dizziness severity, and frequency
Headache	Regulation of basal metabolism and diastolic blood pressure decrease in corticospinal reflex, spinal reflex, and motor excitability improve blood flow to the brain [24]	Myofascial release (MFR) muscle energy high velocity and low amplitude (HVLA), trigger point balanced ligamentous tension (BLT), occipito-atlantal decompression (OA-D), cranial therapy treatment	Decrease in: Headache frequency, intensity, and duration
Inflammation	Increase thoracic duct lymph flow and the flux of protein in thoracic duct lymph decrease in production of nitric oxide, tumor necrosis factor-alpha, and interleukin 10 [25,26]	Lymphatic pump	Reduce inflammation, manage edema
Low back pain	Regulation of positional asymmetry, restricted range of motion, tissue texture abnormalities, or tenderness [7,8]	Spinal manipulation	Improvement in: Pain intensity, pain impact, physical function, health-related quality of life
Musculoskeletal	Decreased circulating tumor necrosis factor-alpha [10]	Unspecified osteopathic manipulative medicine techniques	Reduction in: Physical pain progression of chronic disease
Neurological	Modulation of alpha and gamma motor neuron activity alterations in cutaneous patterns of sympathetic activity. Hypoalgesia effects by segmental inhibition, activation of descending pain inhibitory pathways, and decrease in temporal summation [28]	Spinal manipulation	Pain reduction
Neonatal/Preterm infant disorders	Reduction of pro-inflammatory substances, modulation of parasympathetic and sympathetic actions of the heart [18]	Cranial manipulation	Reduction in length of stay in preterm infants

Limitations

Limitations of this literature mining and analysis include that we set the goal to retrieve only peer-reviewed publications from two public scientific databases, NCBI PubMed and ScienceDirect. We also applied the combinations of chosen keywords and terms to conduct the primary screening. Without searching other bibliographic literature databases or using any manual retrieval strategy, it is possible that publications disseminated through other platforms, for example, journals associated with the field that are not indexed were not retrieved. For readers’ interest, previously Morin and Gaboury had published a comprehensive bibliometric analysis on publications of osteopathic empirical research between 1996 and 2018 [12].

## Conclusions

Based on the findings of this comprehensive literature review, OMT was found to be positive in treating not only some common disorders, for example, pneumonia, low back pain, musculoskeletal disorders, and dizziness, but also other conditions such as anxiety, headache, inflammation, and neonate/preterm infant disorders. Benefits were presented from regulation and healing provided by manual treatments to structure and function. These benefits were based on the four tenets and five models of OM that are interconnected by musculoskeletal anatomy and system. The OMM research in human disorders deserves more government funding and support to advance evidence-based solutions in primary care, family medicine, pediatrics, osteopathic education, and beyond.
